# Clinicopathological characteristics and prognostic analysis of breast cancer with a hormone receptor status of ER(-)/PR(+)

**DOI:** 10.3389/fendo.2023.1193592

**Published:** 2023-07-19

**Authors:** Xinli Wang, Yan Xue

**Affiliations:** Department of Oncology, Xi’an International Medical Center Hospital, Xi’an, China

**Keywords:** breast cancer, hormone receptor, ER(-)/PR(+), nomogram, SEER database

## Abstract

**Background:**

It is unknown whether ER(-)/PR(+) breast cancer is an independent breast cancer subtype, how it differs from other subtypes, and what its significance is regarding treatment and prognosis. This study compared ER(-)/PR(+) breast cancer with other subtypes to better understand the biological characteristics and prognosis of ER(-)/PR(+) breast cancer, to guide clinical treatment and establish a theoretical foundation.

**Methods:**

We retrospectively analyzed data for patients diagnosed with breast cancer in the Surveillance, Epidemiology, and End Results (SEER) database. The clinicopathological characteristics of ER(-)/PR(+) breast cancer, including age, tumor size, lymph node status, HER-2 status, pathological type and histological grade, were compared with other types of breast cancer. A risk scoring system was developed based on independent risk factors influencing prognosis to predict the patient’s prognosis, and a nomogram model was created to predict the patient’s survival rate. Receiver operating characteristic curve (ROC) and calibration curve was used to evaluate the predictive performance of the nomogram.

**Results:**

The rates of T3-4, lymph node positivity, HER-2 positivity, infiltrating non-special pathological type, and G3 were significantly higher in ER(-)/PR(+) than in ER(+)/PR(+) cancer (*p <*0.001). ER(-)/PR(+) was similar to biological activity of ER(-)/PR(-) type. ER(-)/PR(+)/HER-2(+) patients had a better survival prognosis than ER(-)/PR(+) HER-2(-) patients (*p*<0.05). The prognosis of ER-/PR+ breast cancer was significantly associated with age, HER-2 status, and T stage.

**Conclusion:**

ER(-)/PR(+) breast cancer is more similar to ER(-)/PR(-) breast cancer than other breast cancer subtypes, with an early age of onset, a high proportion of infiltrating non-special types, a high histological grade, and a high HER-2 positivity rate. Whether HER-2 positivity can improve the prognosis of ER(-)/PR(+)breast cancer is worth further discussion. The risk scoring system we developed can effectively distinguish between high-risk and low-risk patients. The nomogram we created had a concordance index of 0.736, and the calibration curve showed good agreement between the predicted and observed outcomes.

## Highlights

- This study compared ER(-)/PR(+) breast cancer with other subtypes to better understand the biological characteristics and prognosis of ER(-)/PR(+) breast cancer, guide clinical treatment and establish a theoretical foundation.
- We confirmed the existence of ER(-)/PR(+) tumors. ER (-)/PR (+)type was more aggressive, early onset age, T stage, histological grade is high, It was similar to biological activity of ER(-)/PR(-) type.
- We found that the clinical prognosis of ER(-)/PR(+)/HER-2(+) type breast cancer was improved compared with ER(-)/PR(+)/HER-2(-).
- The nomogram model we created can predict the patient’s prognosis effectively.

## Introduction

Breast cancer is a malignant tumor with highly heterogeneous biological behavior. Breast cancer is divided to different subtypes based on the expression of ER and PR, and the clinical prognoses and treatment strategies for these subtypes are different. Regarding ER(+) and/or PR(+) cancers, the 2010 ASCO/CAP guidance notes that hormone receptor positivity is defined as nuclear immunohistochemical staining for ER and PR that is greater than or equal to 1%, which indicates that the tumor is sensitive to anti-oestrogen therapy, that the tumor is treatable by endocrine therapy, and that the overall prognosis is good. Although both ER and PR are markers that determine response to endocrine therapy, they do not have the same status in terms of importance. ER positivity has been confirmed to be a valuable prognostic marker with ER positive tumors having better survival as compared to ER low and ER negative tumors (1, 2), however, the status of PR and its usefulness as an independent factor in guiding treatment and the decision-making prognosis are still controversial (3–5).

The St. Galen International Breast Cancer Consensus also attaches great importance to the expression of PR. It is believed that a breast cancer can be classified as the luminal A subtype, which indicates a good prognosis and a good response to endocrine therapy, only if PR positivity is ≥ 20%, while cancers with PR <20% are classified as the luminal B subtype, which may be resistant to endocrine therapy and has a poor prognosis.

The proportions of ER(-)/PR(+) breast cancer reported in the literature range from 1% ~ 4% (6, 7). Some studies suggest that this subtype is highly malignant and has strong proliferation ability (8), but other studies suggest that this subtype does not exist and is a result of detection errors (9). Thus, is ER(-)/PR(+) a unique phenotype? Is there a biological behavioral difference between the ER(+)/PR(-) type and ER(-)/PR(-) type? What is the significance in terms of treatment and prognosis? These questions are worth exploring.

The purpose of this study was to research the clinicopathological characteristics and prognosis of ER(-)/PR(+) breast cancer, and construct a nomogram to effectively predict the prognosis of patients, and provide a basis for developing more suitable individualized treatment strategies. The clinicopathological data of 35154 breast cancer patients from 2010 to 2013 in the US SEER database were collected.

## Patients and methods

### General information

This was a retrospective analysis of the data of patients diagnosed with breast cancer obtained from the SEER database from 2010 to 2013. These data included age, hormone receptor status, tumor size, lymph node status, HER-2 status, pathological type and histological grade. The inclusion criteria were as follows: (1) histopathologicaly confirmed case of breast cancer; (2) females with clinical stage T1~4 N0~3 M0 breast cancer; (3) complete clinicopathological data including age, stage, hormone receptor status, HER-2 status, pathological type and histological grade; (4) patients with surgery and postoperative systemic treatment. The exclusion criteria were bilateral breast cancer, male breast cancer, advanced breast cancer, and patients with incomplete pathological characteristics. 35154 patients were selected for follow-up analysis. The identifiable information is not contained in the public databases, so the approval of an ethics committee was not required. The ethics committee of Xi’an International Medical Center Hospital waived the need for patient consent, and the reference number was 2022095.

### Statistical analysis

SPSS20.0 statistical software was used for data processing. Chi-square test was used to compare groups. The Kaplan-Meier method was used for survival analysis, and the log-rank method was used for pairwise comparisons between groups. Binary logistic regression and Cox proportional hazards regression were used to analyze the independent risk factors affecting the prognosis of ER-/PR+ breast cancer patients. Kaplan-Meier was used to draw survival curves. The median value of probability values of joint variables (i.e., linear prediction values) was used as the cutoff to classify patients into high-risk and low-risk groups. The reliability of the model was verified using ROC and calibration curve. The test level α=0.05.

## Results

### Clinicopathological characteristics of ER(-)/PR(+) patients

In this study, a total of 35154 patients were enrolled, including 352 cases (1.0%) of ER(-)/PR(+), 3819 cases (10.86%) of ER(+)/PR(-), 25622 cases (72.89%) of ER(+)/PR(+), and 5361 cases (15.25%) of ER(-)/PR(-). Compared with ER(+)/PR(+) type, the ER(-)/PR(+) type has statistical differences in age of onset, T stage, N stage, HER-2 status, pathological type, and histological grade, with younger age of onset (χ2 = 333.115, *p <*0.001), higher T stage (χ2 = 101.792, *p <*0.001), higher N stage (χ2 = 10.928, *p <*0.001), higher HER-2 positive rate (χ2 = 92.584, *p <*0.001), higher proportion of non-special pathological type (χ2 = 29.222, *p <*0.001), and higher histological grade (χ2 = 777.194, *p <*0.001). Compared with ER(+)/PR(-) type, the ER(-)/PR(+) type has differences in age of onset (χ2 = 39.719, *p <*0.001), T stage (χ2 = 18.545, *p <*0.001), pathological type (χ2 = 20.603, *p <*0.001), and histological grade (χ2 = 202.543, *p <*0.001), with younger age of onset, higher T stage, higher proportion of non-special pathological type, and higher histological grade. There were no statistical differences in age of onset, T stage, N stage, HER-2 status, pathological type, and histological grade between ER(-)/PR(+) and ER(-)/PR(-) types (*p >*0.05) (**Tables 1**, **2**).

**Table 1:** Clinicopathological characteristics (n,%).

group	group(n,%)				χ2 value	P value
	ER(-)/PR(+)	ER(+)/PR(-)	ER(+)/PR(+)	ER(-)/PR(-)		
age (years)						
<35	10(2.84)	73(1.9)	319(1.2)	163(3.0)	χ2 = 347.962	P<0. 001
35-59	201(57.10)	1551(40.6)	11020(43.0)	2878(53.7)		
≥60	141(40.06)	2195(57.5)	14283(55.7)	2320(43.3)		
tumor size						
T1	168(47.7)	2252(59.0)	17956(70.1)	2459(45.9)	χ2 = 1344.368	P<0. 001
T2	136(38.6)	1166(30.5)	6034(23.6)	2082(38.8)		
T3	27(7.7)	261(6.8)	1205(4.7)	512(9.6)		
T4	21(6.0)	140(3.7)	427(1.7)	308(5.7)		
lymph node status						
negtive	220(62.5)	2507(65.5)	18087(70.6)	3215(60.0)	χ2 =252.030	P<0. 001
positive	132(37.5)	1312(34.4)	7535(29.4)	2146(40.0)		
HER-2 status						
negtive	269(76.4)	3045(79.7)	23367(91.2)	4091(76.3)	χ2 =1187.138	P<0. 001
positive	83(23.6)	774(20.3)	2255(8.8)	1270(23.7)		
pathological type						
non-special Infiltrating	309(87.8)	2954(77.4)	19294(75.3)	4682(87.3)	χ2 = 389.499	P <0. 001
others	43(12.2)	865(22.6)	6328(24.7)	679(12.7)		
histological grade						
grade I	7(2.0)	694(18.2)	7640(29.8)	109(2.0)	χ2 = 6827.256	P <0. 001
grade II	63(17.9)	1547(40.5)	12884(50.3)	1000(18.7)		
grade III	282(80.1)	1578(41.3)	5098(19.9)	4252(79.3)		

**Table 2:** Comparison results of clinicopathological data of 4 groups of patients.

Comparison between groups	χ2 value	P value
age		
ER(-)/PR(+)vs ER(+)/PR(-)	χ2 = 39.719	P<0. 001
ER(-)/PR(+)vs ER(+)/PR(+)	χ2 = 333.115	P<0. 001
ER(-)/PR(+)vs ER(-)/PR(-)	χ2 =1.891	P=0.413
ER(+)/PR(-)vs ER(+)/PR(+)	χ2 =17.339	P=0.247
ER(+)/PR(-)vs ER(-)/PR(-)	χ2 =181.478	P <0. 001
tumor size		
ER(-)/PR(+)vs ER(+)/PR(-)	χ2 = 18.545	P<0. 001
ER(-)/PR(+)vs ER(+)/PR(+)	χ2 = 101.792	P<0. 001
ER(-)/PR(+)vs ER(-)/PR(-)	χ2 =1.517	P=0.688
ER(+)/PR(-)vs ER(+)/PR(+)	χ2 =225.334	P <0. 001
ER(+)/PR(-)vs ER(-)/PR(-)	χ2 =157.352	P <0. 001
lymph node status		
ER(-)/PR(+)vs ER(+)/PR(-)	χ2 =1.409	P=0.242
ER(-)/PR(+)vs ER(+)/PR(+)	χ2 = 10.928	P<0. 001
ER(-)/PR(+)vs ER(-)/PR(-)	χ2 =0.882	P=0. 369
ER(+)/PR(-)vs ER(+)/PR(+)	χ2 =38.683	P <0. 001
ER(+)/PR(-)vs ER(-)/PR(-)	χ2 =30.595	P <0. 001
HER-2 status		
ER(-)/PR(+)vs ER(+)/PR(-)	χ2 =2.166	P=0.148
ER(-)/PR(+)vs ER(+)/PR(+)	χ2 = 92.584	P <0. 001
ER(-)/PR(+)vs ER(-)/PR(-)	χ2 =0.002	P=1.000
ER(+)/PR(-)vs ER(+)/PR(+)	χ2 =473.416	P <0. 001
ER(+)/PR(-)vs ER(-)/PR(-)	χ2 =15.094	P <0. 001
pathological type		
ER(-)/PR(+)vs ER(+)/PR(-)	χ2 = 20.603	P <0. 001
ER(-)/PR(+)vs ER(+)/PR(+)	χ2 = 29.222	P <0. 001
ER(-)/PR(+)vs ER(-)/PR(-)	χ2 =0.060	P=0.442
ER(+)/PR(-)vs ER(+)/PR(+)	χ2 =7.548	P=0.006
ER(+)/PR(-)vs ER(-)/PR(-)	χ2 =158.915	P <0. 001
histological grade		
ER(-)/PR(+)vs ER(+)/PR(-)	χ2 = 202.543	P <0. 001
ER(-)/PR(+)vs ER(+)/PR(+)	χ2 = 777.194	P <0. 001
ER(-)/PR(+)vs ER(-)/PR(-)	χ2 =0.164	P=0.961
ER(+)/PR(-)vs ER(+)/PR(+)	χ2 =897.365	P <0. 001
ER(+)/PR(-)vs ER(-)/PR(-)	χ2 =1555.906	P <0. 001

### Comparison of overall survival curves of 4 types groups of patients 

**Figure 1:**
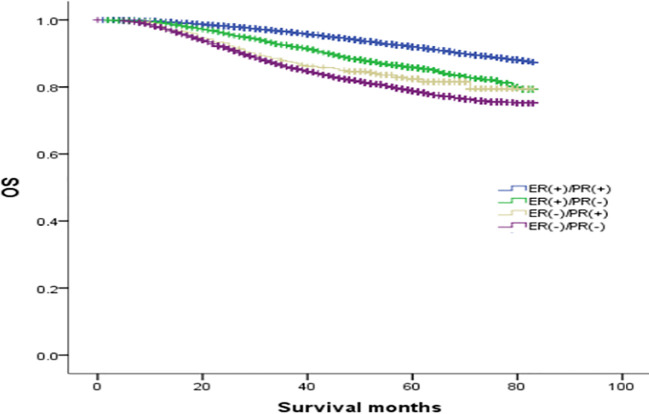
Comparison of overall survival curves of 4 types groups of patients.

The median follow-up was 64 months, and the OS rates of patients with ER(+)/PR(+), ER(+)/PR(-), ER(-)/PR(+), and ER(-)/PR(-) types were 91.1%, 84.6%, 82.1%, and 78.2%, respectively (χ2 = 870.695, *p <*0.001). Among them, the ER(+)/PR(+) type had the best prognosis, and the OS differences were statistically significant compared with the ER(+)/PR(-), ER(-)/PR(+), and ER(-)/PR(-) types (χ2 = 167.172, *p <*0.001; χ2 = 41.525, *p <*0.001; χ2 = 834.420, *p <*0.001). There were no statistically significant differences in OS between the ER(-)/PR(+) type and the ER(+)/PR(-) or the ER(-)/PR(-) type (χ2 = 2.468, *p* =0.116; χ2 = 2.528, *p* =0.112), but there was a statistically significant difference in OS between the ER(+)/PR(-) type and the ER(-)/PR(-) type (χ2 = 65.536, *p <*0.001). In summary, the prognosis of the four types in order from best to worst was: ER(+)/PR(+), ER(+)/PR(-), ER(-)/PR(+), and ER(-)/PR(-) (**Figure 1**).

### The prognosis and survival analysis of ER(-)/PR(+) patients based on HER2 status

**Figure 2:**
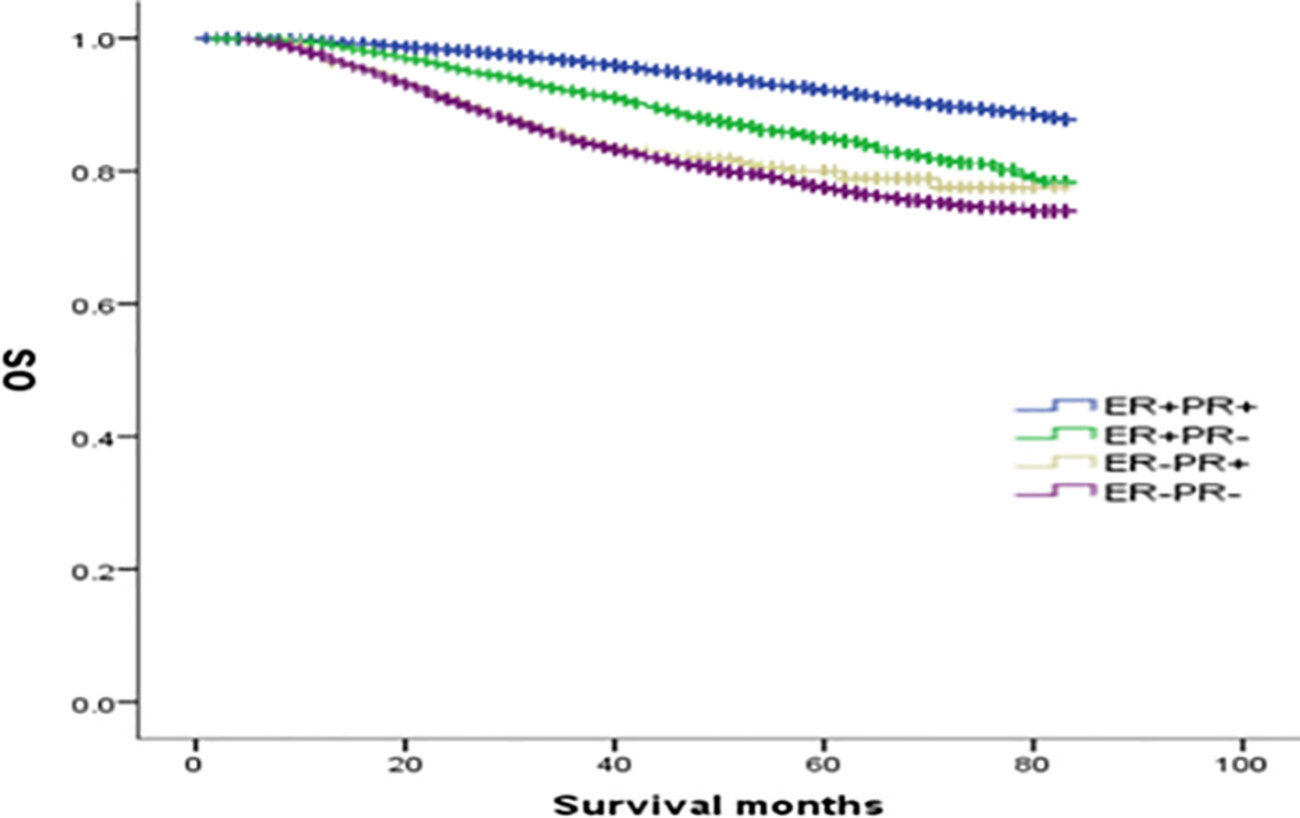
Comparison of overall survival curves of 4 types groups of patients with HER-2(-) breast cancer (χ2 = 899.206, *p*<0.001). Among them, ER(-)/PR(+) type and ER(-)/PR(-) type have no significant difference (χ2 = 0.725, *p* =0.395), and there are statistical differences between the other types, ER(-)/PR(+) and ER(+)/PR(-) type (χ2 = 4.423, *p* =0.035); ER(-)/PR(+) type and ER(+)/PR(+) type (χ2 = 55.794, *p*<0.001); ER(+)/PR(-) type and ER(-)/PR(-) type (χ2 = 54.622, *p*<0.001).

**Figure 3:**
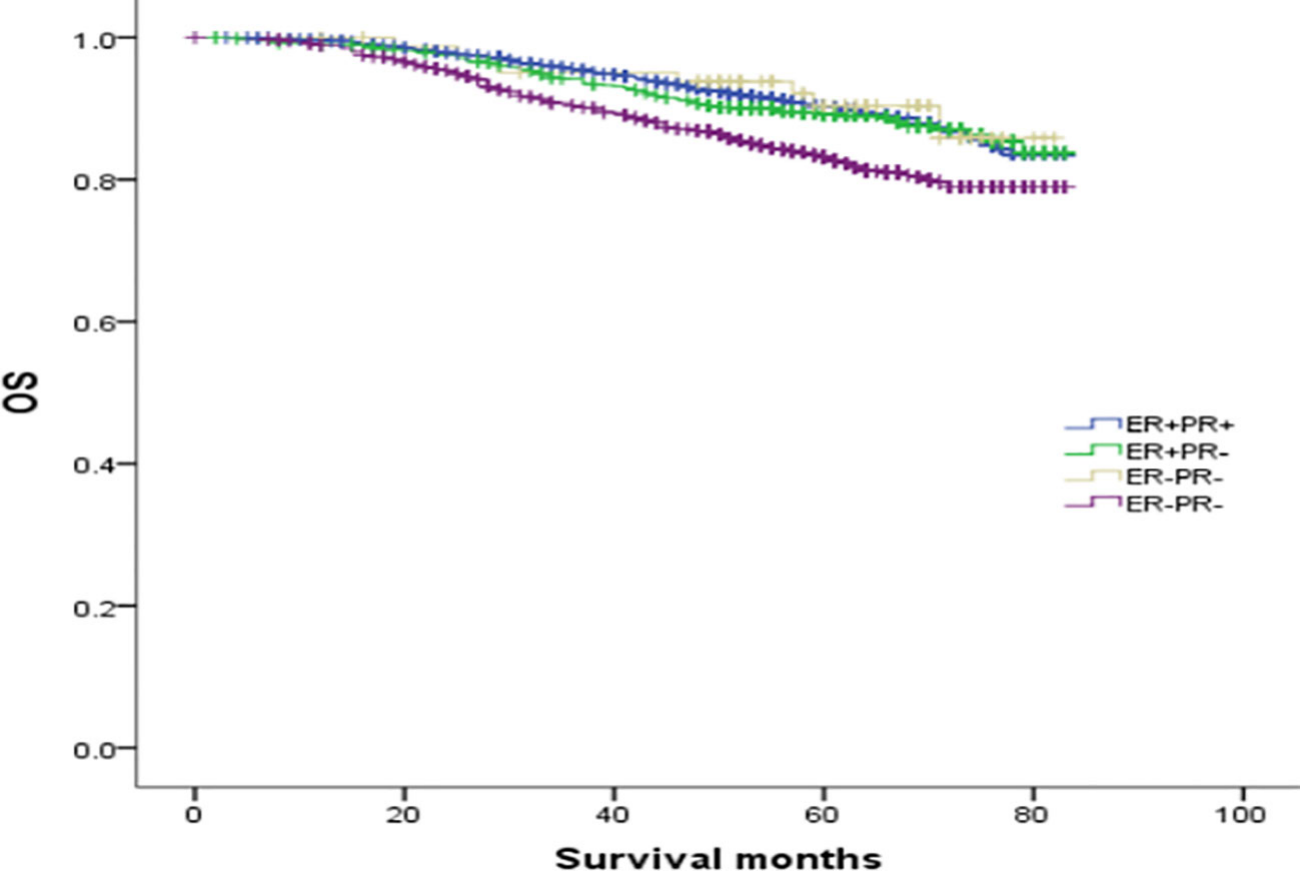
Comparison of overall survival curves of 4 types groups of patients with HER-2(+) breast cancer (χ2 = 38.339, *p*<0.001). Among them, ER(-)/PR(+) type and ER(+)/PR(-) type (χ2 = 0.253, *p* =0.615); ER(-)/PR(+) type and ER(-)/PR (-) type (χ2 = 3.444, *p* =0.063); ER(-)/PR(+) type and ER(+)/PR(+) type (χ2 = 0.099, *p* =0.752); ER(+)/PR(-) and ER(+)/PR(+) type (χ2 = 0.253, *p* =0.615); ER(+)/PR(-) type and ER(-)/PR(-) type (χ2 = 14.592), *p*<0.001); ER(+)/PR(+) type and ER(-)/PR(-) type (χ2 = 34.598, *p*<0.001).

To explore the effect of HER-2 status on the prognosis of breast cancer with different ER and PR phenotypes, further analysis of four types of hormone receptor phenotypes was carried out according to HER-2(-) and HER-2(+) status. In total, there were 30772 cases (87.53%) of HER-2(-) breast cancer: 269 ER(-)/PR(+) cases (0.87%), 3045 ER(+)/PR(-) cases (9.9%), 23367 ER(+)/PR(+) cases (75.94%), and 4091 ER(-)/PR(-) cases (13.29%). There were 4382 cases (12.47%) of HER-2(+) breast cancer: 83 ER(-)/PR(+) cases (1.89%), 774 ER(+)/PR(-) cases (17.66%), 2255 ER(+)/PR(+) cases (51.46%), and 1270 ER(-)/PR(-) cases (28.98%) (see **Table 3** for details). In the HER-2(-) subgroup, at a median follow-up of 64 months, the overall survival (OS) in the ER(-)/PR(+), ER(+)/PR(-) and ER(+)/PR(+) groups were 79.6%, 83.6%, 91.3% and 76.9%, respectively. Compared with the 4 groups of patients, the difference was statistically significant (χ2 = 899.206, *p <*0.001). Specifically, the difference between the ER(-)/PR(+) type and ER(-)/PR(-) type was not significant (χ2 = 0.725, *p* =0.395), but there were significant differences between the other groups, as shown in **Figure 2**. In the HER-2(+) subgroup, the OS in the ER(-)/PR(+), ER(+)/PR(-) and ER(+)/PR(+) group were 77.43%, 77.17%, 77.8% and 74.08%, respectively. Similarly, compared with the 4 groups of patients, the difference was statistically significant (χ2 = 38.339, *p <*0.001). Among the groups, there was no significant difference between the ER(-)/PR(+) group and the ER(+)/PR(-), ER(-)/PR(-), or ER(+)/PR(+) group (χ2 = 0.253, *p* = 0.615; χ2 = 3.444, *p* =0.063; χ2 = 0.099, *p* =0.752). In addition, there was no significant difference between the ER(+)/PR(-) and ER(+)/PR(+) group (χ2 = 0.253, *p* =0.615) (**Figure 3**).

**Table 3:** Proportion of 4 groups of patients in different HER-2 status.

HER-2 status	cases	group(n,%)			
		ER(-)/PR(+)	ER(+)/PR(-)	ER(+)/PR(+)	ER(-)/PR(-)
HER-2(-)	30772(87.53%)	269(0.87%)	3045(9.9%)	23367(75.94%)	4091(13.29%)
HER-2(+)	4382(12.47%)	83(1.89%)	774(17.66%)	2255(51.46%)	1270(28.98%)

**Figure 4:**
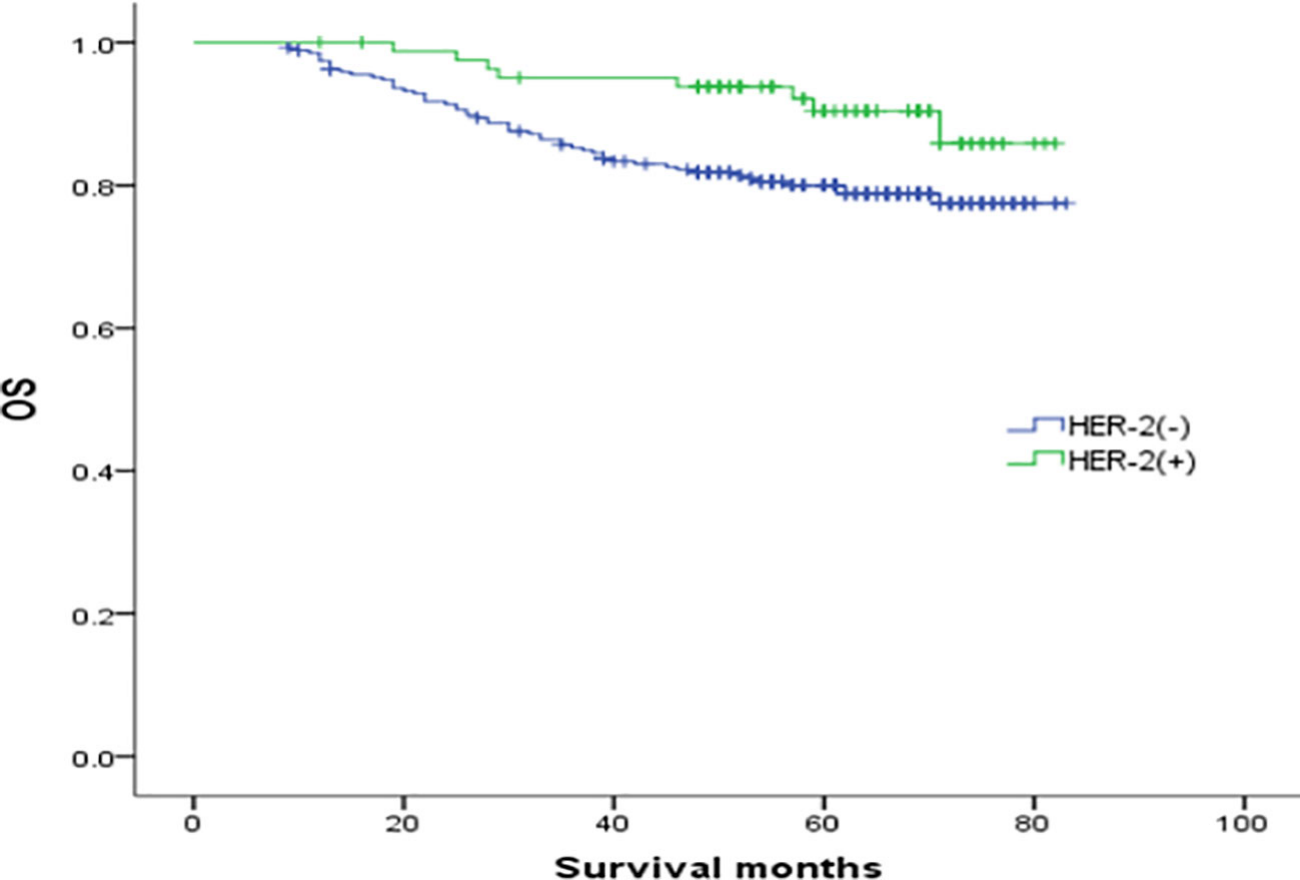
Comparison of overall survival curves of ER(-)/PR(+)type breast cancer with different HER-2 status (χ2 = 5.021, *p* =0.025).

**Figure 5:**
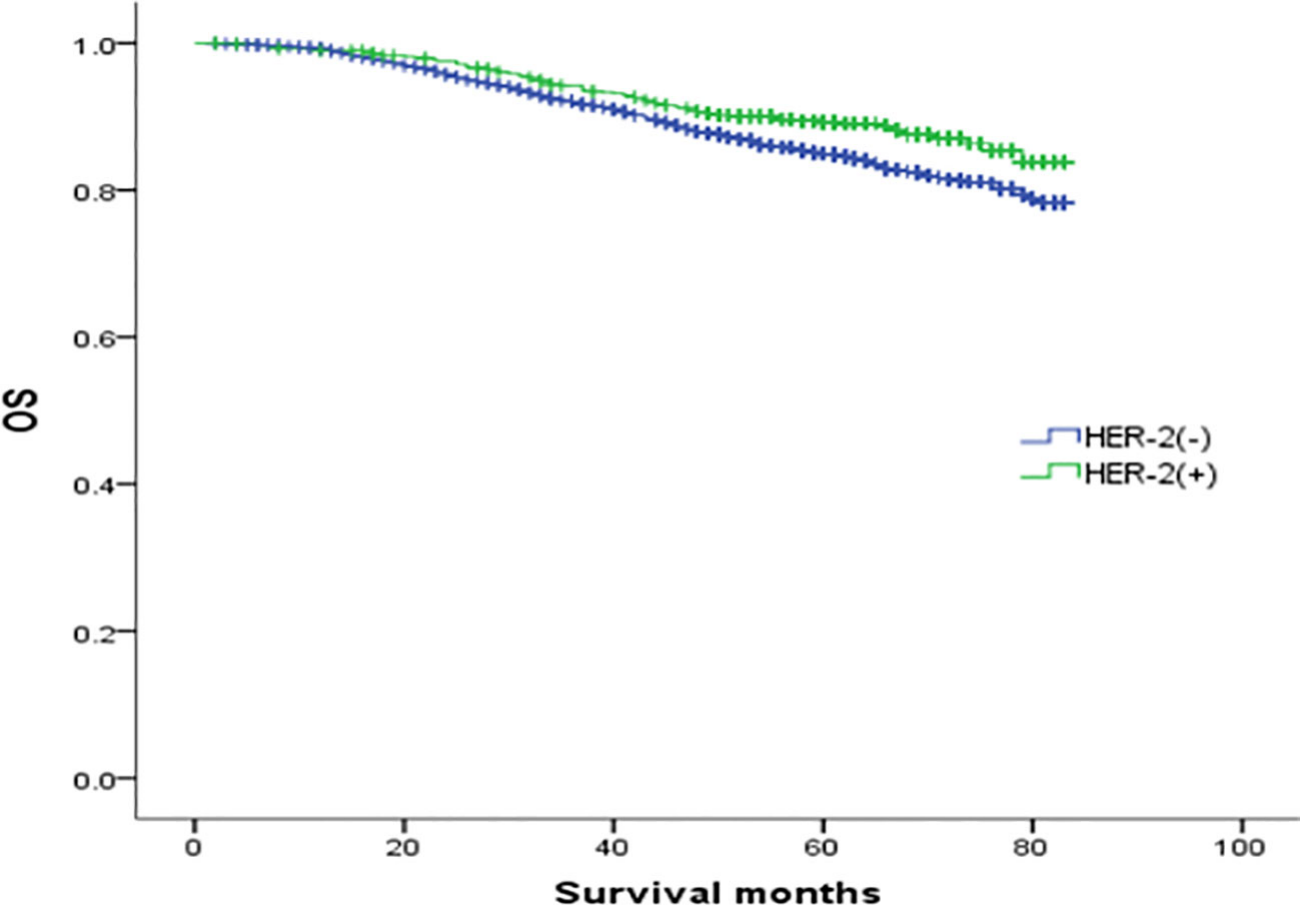
Comparison of overall survival curves of ER(+)/PR(-) phenotype breast cancer with different HER-2 status (χ2 = 10.018, *p* =0.002).

**Figure 6:**
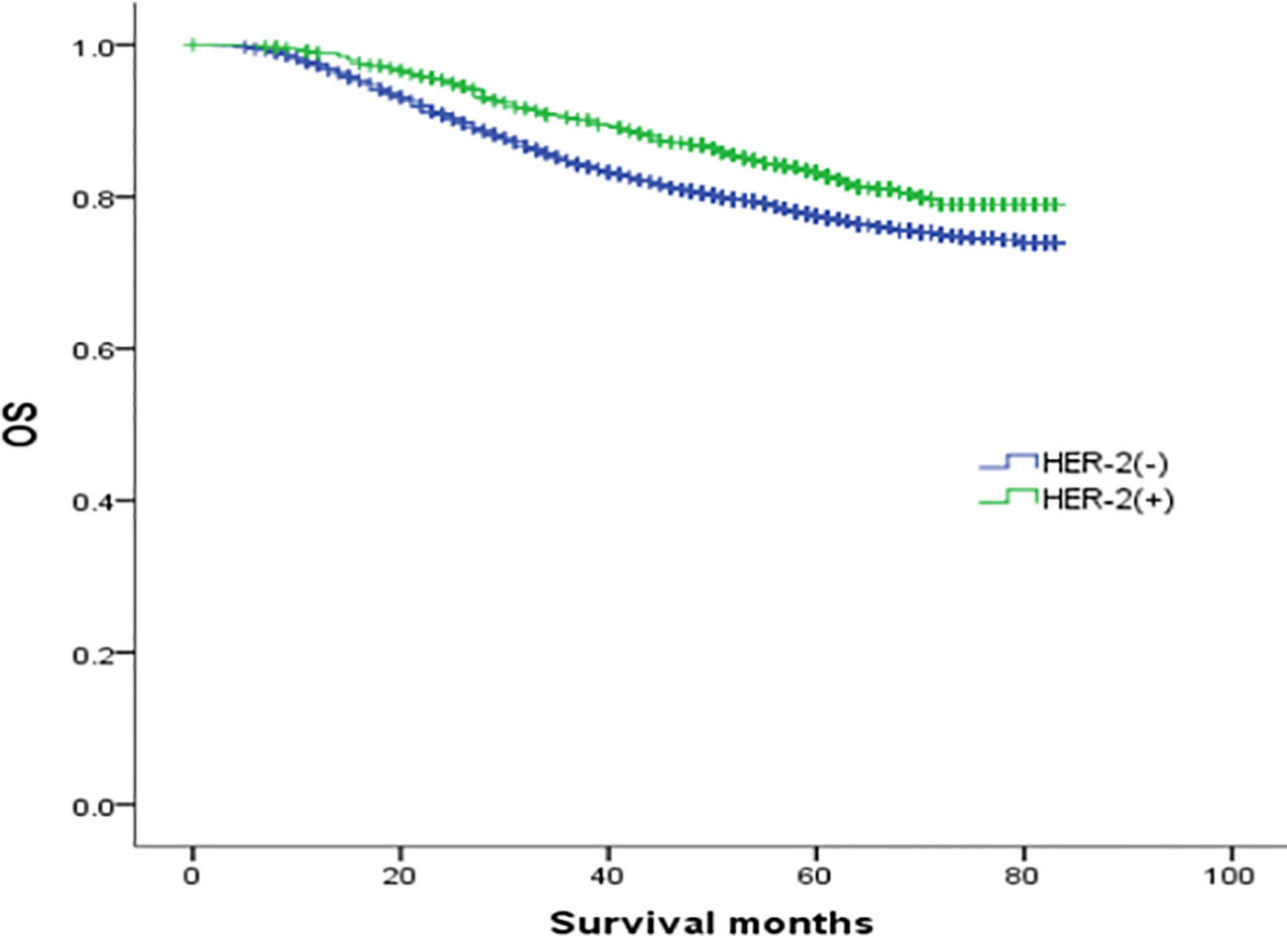
Comparison of overall survival curves of ER(-)/PR(-) phenotype breast cancer with different HER-2 status (χ2 = 16.247, *p*<0.05).

**Figure 7:**
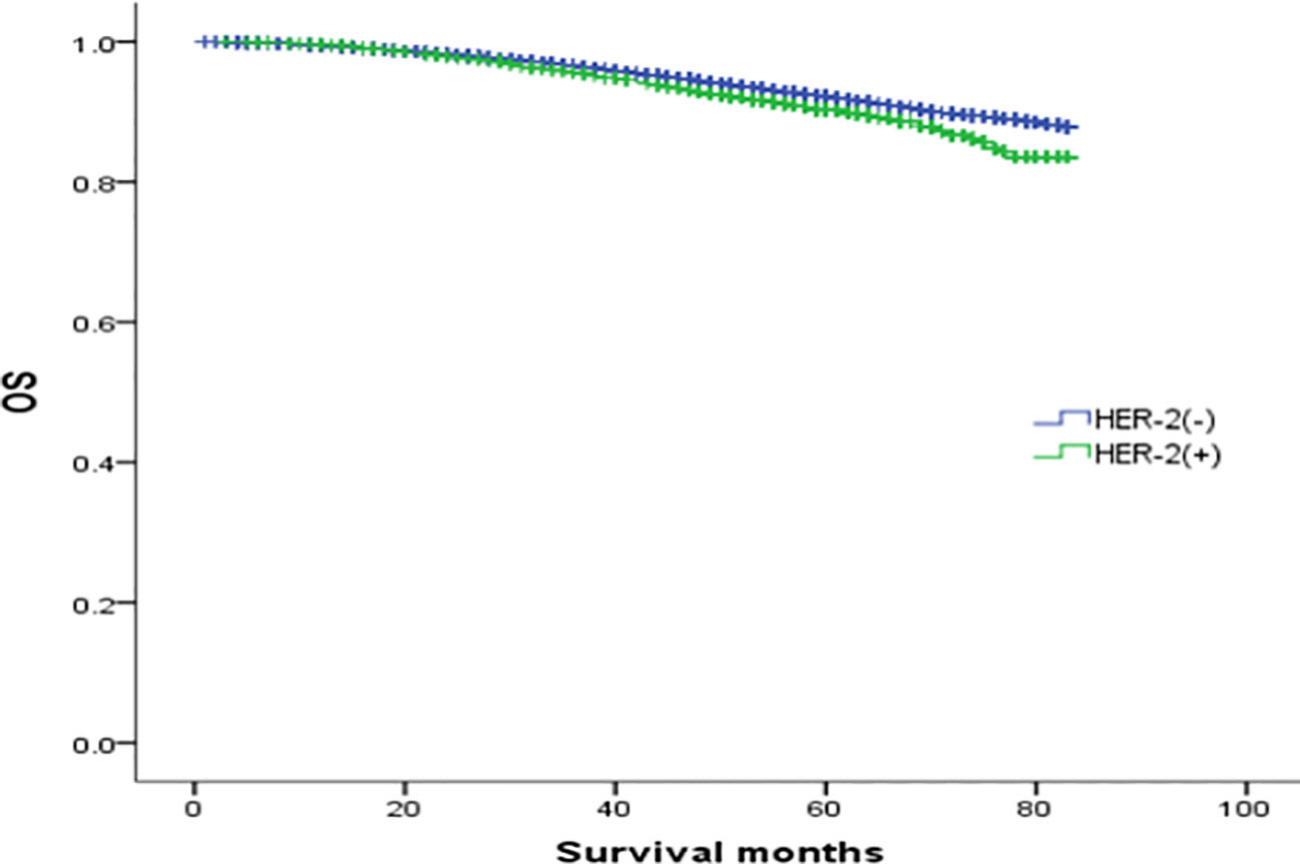
Comparison of overall survival curves of ER(+)/PR(+)phenotype breast cancer with different HER-2 status (χ2 = 14.748, *p*<0.05).

**Figure 8:**
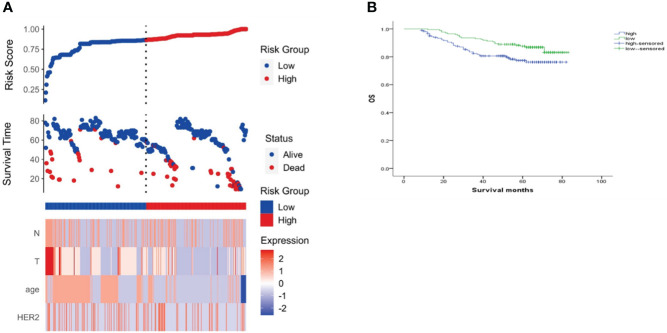
Risk scoring model. **(A)** Risk score distribution, survival status, and heat map of risk factor markers; **(B)** K-M curve of the high/low-risk group.

In the analysis of HER-2 subgroups according to hormone receptor status, the survival prognosis of ER(-)/PR(+)/HER-2(+) patients was improved compared with that of ER(-)/PR(+)/HER-2(-) patients (χ2 = 5.021, *p* < 0.05) (**Figure 4**); similarly, HER-2 status of ER(+)/PR(-) and ER(-)/PR(-) affects their prognosis, HER-2(+) patients have better prognosis. However, ER(+)/PR(+) HER-2(-) patients had a better prognosis than ER(+)/PR(+) HER-2(+) (χ2 = 14.748, *p <*0.05) (**Figures 5**–**8**).

### Development and validation of a nomogram based on clinical risk factors

**Figure 9:**
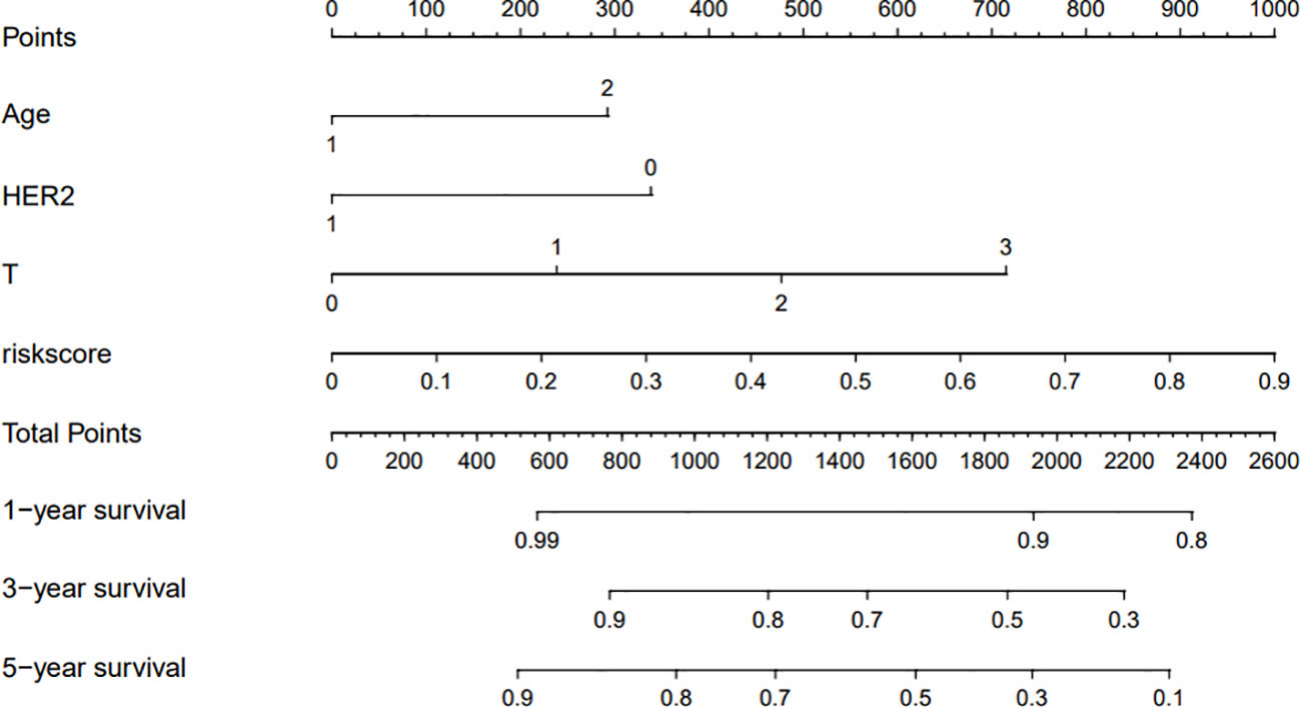
Nomogram to predict 1/3/5-year overall survival of ER-/PR+ breast cancer patients.

**Figure 10:**
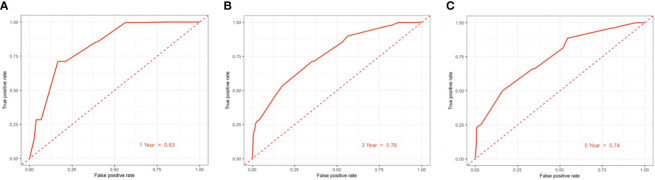
ROC curve for predicting 1/3/5-year overall survival of ER-/PR+ patients with the nomogram. **(A)** 1-year overall survival; **(B)** 3-year overall survival; **(C)** 5-year overall survival.

**Figure 11:**
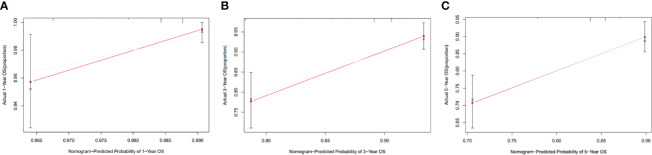
Calibration curve of the nomogram for 1-, 3-, and 5-year OS probability(C-index = 0.736). **(A)** 1-year OS probability; **(B)** 3-year OS probability; **(C)** 5-year OS probability.

The clinicopathological factors related to the prognosis of ER-/PR+ patients were screened by univariate and multivariate analysis (**Table 4**). A nomogram was constructed (**Figure 9**), and the AUC values for 1-year, 3-year, and 5-year survival were 0.83, 0.76, and 0.74, respectively (**Figure 10**). The bootstrap method was used for internal validation with 1,000 bootstrap samples, and the C-index was 0.736, indicating good discrimination of the model. Calibration curves were plotted for 1-year, 3-year, and 5-year survival rates, and the results showed good agreement between the actual and predicted survival rates, indicating good calibration and accuracy of the model (**Figure 11**).

**Table 4:** Univariate and multivariate analysis of ER-/PR+ patients.

Factors	*P* value	
	Univariate analysis	multivariate analysis
Age (y)	0.005	0.003
HER2 status	0.008	0.003
Pathological type	0.109	
Histological grade	0.628	
T stage	0.001	0.001
N stage	0.025	

## Discussion

PR is a complex intracellular receptor and a member of the nuclear receptor family of ligand-dependent transcription factors. Its main role is to regulate the expression of target genes (10, 11). Studies have shown that the expression level of PR is affected by ER, and PR can be used as an indicator of a fully functional nuclear ER pathway, helping to predict which patients will respond to hormone therapy (12–16).

Among single-positive hormone receptor patients, the proportion of PR-positive patients alone is very small, at approximately 1% to 4% in the literature. Due to the low incidence, some scholars in the past suggested that it may only be the product of laboratory test errors. For patients with negative ER cancers, PR status has no independent prognostic value (17). Foley et al. performed repeated tests on 56 specimens of ER(-)/PR(+) breast cancer diagnosed by immunohistochemistry, and none of them was a real ER(-)/PR(+) breast cancer (18). However, according to clinical manifestations and global genotypic data analysis, ER(-)/PR(+) breast cancer is a unique breast cancer subtype (19). In practice, even in the best fixed tissue and with any level of tumor cell nuclear immunoreactivity being used as a positive result, ER(-)/PR(+) remains a unique entity (9, 20). The proportion of ER(-)/PR(+) in this study is 1%, which confirms that ER(-)/PR(+) tumors do exist.

Since it was first proposed in 1975, that PR expression can predict the prognosis of advanced disease and the response to ER-guided treatment, the potential utility of PR expression as a prognostic marker has been recognized (21). This was later confirmed in a prospective study (22). The expression of PR predicts the response of premenopausal women to tamoxifen, and as the expression level of PR increases, the response increases (23). PR expression is a surrogate marker of a functional ER pathway. Therefore, patients whose tumors have ER(-)/PR(+) phenotypes can still achieve survival benefits from hormone therapy. In many studies, the independent prognostic value of PR has been explored in ER-positive early and advanced breast cancer (24–26). These studies strongly support the notion that PR status not only is related to ER function but also reflects ER interacting with growth factor signaling. PR deficiency may lead to estrogen receptor pathway block and resistance to endocrine therapy (27–30).

The findings consistently indicate that ER(+)/PR(+) had the best prognosis, and ER(-)/PR(-) had the worst prognosis, while the ER(+)/PR(-) group is somewhere between them. Regarding the prognostic significance of ER(-)/PR(+) tumors, due to the rarity of this subtype, there are few studies. This study shows that ER(-)/PR(+) breast cancer has higher rates of T3-4 stage, invasive non-special pathological type, and a high G3 ratio than ER(+)/PR(+) or ER(+)/PR(-) breast cancer, where there are significant differences, but these rates are not significantly different from those of ER(-)/PR(-) cancer. However, the rates of lymph node metastasis and HER-2 positivity were not significantly different from those of ER(-)/PR(-) cancer, and there were no significant differences in these rates compared with ER(+)/PR(-) cancer. These results suggest that compared with ER(+)/PR(-) and ER(+)/PR(+) breast cancer, ER(-)/PR(+) breast cancer is more aggressive and has an earlier age of onset. The T stage is poor, the histological grade is high, and the biological activity is similar to that of ER(-)/PR(-). Interestingly, there was no significant survival difference between ER(-)/PR(+) and ER(+)/PR(-) breast cancers, and between ER(-)/PR(+) and ER(-)/PR(-).

In this study, we found that ER(-)/PR(+) breast cancer had higher levels of HER2. In the HER-2(-) subgroup, ER(-)/PR(+) tumor patients and ER(+)/PR(+) tumor patients had higher prognostic differences, and ER(-)/PR(+) clinical outcomes were worse. In the HER-2(+) subgroup, the clinical outcome of ER(-)/PR(+) had a trend toward improvement, which may have a better prognosis than breast cancer with other hormone receptor phenotypes, indicating that ER(-)/PR(+)/HER-2(-) tumors are aggressive, if combined with HER-2(+), the clinical prognosis can be improved. The reason may be that the efficacy of endocrine therapy in these patients is poor, but if the cancer is HER-2 (+), patients may benefit from anti-HER-2 therapy. The specific reasons need to be further explored. In addition, we found that ER(+)/PR(-) tumors have the same characteristics, which indicates that even if ER(+)/PR(-) tumors are HER-2 negative, their prognosis is also poor, and the survival prognosis is not as good as that of patients with HER-2(+) tumors, which also supports the hypothesis that PR expression is an independent prognostic factor for breast cancer.

The results show that PR expression is an independent prognostic factor of breast cancer. The clinical features and prognosis of ER(-)/PR(+) breast cancer are similar to those of ER(-)/PR(-).The characteristics of a high T3-4 stage ratio, a high proportion of non-special invasive types, a high histological grade, and a high HER-2 positive rate indicate that this subtype represents a group of hormone receptor-positive tumors with high malignancy and poor prognosis. In the absence of ER expression, the reason for PR expression is still unclear, and further research in this case is necessary. ER(-)/PR(+) breast cancer forms independent subtypes with unique molecular and clinical characteristics, and overall gene expression data also support the existence of an invasive ER(-)/PR(+) breast cancer phenotype. Some studies categorized ER(-)/PR(+) by PAM50 molecular typing, and the vast majority of cases were classified as triple-negative type (53%-65%), followed by luminal type (15%-27%), and luminal subtypes were shown to be more sensitive to endocrine therapy than triple-negative type patients (25, 31, 32). This indicates that most ER(-)/PR(+) breast cancers have the molecular characteristics of triple negative breast cancer cases, which is also the reason why ER(-)/PR(+) has similar clinical features and prognosis to ER(-)/PR(-).

In this study, although the prognosis of ER(-)/PR(+) and ER(-)/PR(-) was similar, it was not entirely consistent. In **Figure 1**, we can see that the survival curves of ER(-)/PR(+) and ER(-)/PR(-) do not coincide, and with the extension of follow-up time, the survival curves of ER(-)/PR(+) and ER(-)/PR(-) become more and more separated, and we even speculate that if the follow-up time is extended, ER(-)/PR(+) may obtain survival results with statistically significant differences compared with ER(-)/PR(-). In **Figure 2**, we can see that the survival curves of ER(-)/PR(+) and ER(-)/PR(-) are very close among HER-2 negative patients, but if HER-2 positive, the survival curves of ER(-)/PR(+) and ER(-)/PR(-) are clearly separated. Therefore, we believe that the biological behavior and prognosis of ER(-)/PR(+) and ER(-)/PR(-) are different, and ER(-)/PR(+) is a distinct molecular subtype of breast cancer from ER(-)/PR(-). Therefore, the existence of ER(-)/PR(+) breast cancer phenotype was verified from the perspective of clinical prognosis and overall expression data of breast cancer.

This study established a prediction model based on data from ER(-)/PR(+) patients in the SEER database, identified independent risk factors for the prognosis of ER(-)/PR(+) patients, and demonstrated the model's ability to accurately and effectively predict their prognosis. This can provide a scientific basis for clinical treatment.

## Conclusion

We analyzed the biological behavior and survival of different types of breast cancer in the SEER database, and confirmed the existence of ER(-)/PR(+) tumors. ER (-)/PR (+)type compared to ER(+)/PR(-)、ER(+)/PR(+) breast cancer, more aggressive, early onset age, T stage, histological grade is high, It was similar to biological activity of ER(-)/PR(-) type. We found that if ER(-)/PR(+) type breast cancer is combined with HER-2(+), the clinical prognosis is improved compared with HER-2(-), suggesting that these patients have poor effect of endocrine therapy, but if HER-2(+), they may benefit from anti-HER2 treatment. In addition, we found that ER(+)/PR(-) had the same characteristics. The results suggest that PR expression is an independent prognostic factor of breast cancer. These findings enable us to better understand the biological characteristics and prognosis of ER(-)/PR(+) breast cancer, and lay a theoretical foundation for clinical treatment. In this study, the proportion of ER-/PR+ was only 1%, and the number was small, so it is necessary to increase the sample size and further increase the follow-up time. We constructed a nomogram to effectively predict the prognosis of ER(-)/PR(+) patients, providing a basis for more suitable individualized treatment strategies.

## Data availability statement

The original contributions presented in the study are included in the article/supplementary material. Further inquiries can be directed to the corresponding author.

## Author contributions

XW designed the study, collected the data, and wrote the paper. YX contributed to the conception and design of the study, wrote sections of the manuscript, and revised it critically. All authors contributed to the article and approved the submitted version.
